# A Review and Experimental Study on the Performance of Rubberised Concrete Under Combined Freeze–Thaw and Sulphate Attack

**DOI:** 10.3390/ma19051011

**Published:** 2026-03-06

**Authors:** Josep Ramon Lliso-Ferrando, Pablo Márquez-Gómez, José Manuel Gandía-Romero, Manuel Valcuende

**Affiliations:** 1 Research Institute for Molecular Recognition and Technological Development (IDM), Universitat Politècnica de València, Camino de Vera, s/n., 46022 Valencia, Spain; joganro@csa.upv.es; 2 Department of Architectural Constructions, School of Architecture, Universitat Politècnica de València, Camino de Vera, s/n., 46022 Valencia, Spain; mvalcuen@csa.upv.es; 3 School of Architecture, Universitat Politècnica de València, Camino de Vera, s/n., 46022 Valencia, Spain; pabma18p@arq.upv.es

**Keywords:** rubberised concrete, review, recycled rubber, end-of-life tyres, sand replacement, durability, freeze–thaw, sulphate attack

## Abstract

The use of end-of-life tyre (ELT) rubber as a partial aggregate replacement in concrete represents a promising route for waste valorisation; however, its durability-related behaviour and long-term performance remain insufficiently characterised, particularly under combined environmental exposures. This study addresses these limitations by combining a targeted literature review encompassing more than 4500 data points from over 150 published studies with a laboratory-based experimental assessment of rubberised concretes aimed at clarifying key knowledge gaps. The experimental programme investigates concretes incorporating 5–50% ELT rubber (0/4 mm) as a selective replacement of a specific sand fraction, rather than of the total fine aggregate content, with particular emphasis on performance under coupled freeze–thaw cycling and sulphate attack. A reference mix (>50 MPa at 28 days) and seven rubberised concretes were characterised in terms of mechanical behaviour and selected durability-related indicators. Specimens were subsequently exposed for 270 days to freeze–thaw cycles (−20/+20 °C) in a 10% MgSO_4_ solution, and surface damage and compressive strength loss were quantified. Increasing rubber content resulted in the expected reductions in mechanical performance, accompanied by lower electrical resistivity and increased porosity and carbonation depth. However, the selective replacement of a single sand fraction led to more gradual deterioration than typically reported for global sand substitution. Under combined freeze–thaw and sulphate exposure, concretes with low rubber contents (5–15%) exhibited no observable surface damage and retained most of their mechanical capacity, with compressive strength losses below 8%, whereas mixtures with ≥30% replacement showed pronounced surface degradation and strength losses exceeding 50%.

## 1. Introduction

Concrete currently underpins the development of buildings and civil infrastructure worldwide [1,2], with annual production estimated at tens of gigatonnes (around three times the volume produced 40 years ago) and demand rising more rapidly than for steel or wood [3]. Its success stems from a unique combination of mechanical properties, durability and versatility, coupled with relatively low production costs and the widespread availability of its constituents [4]. Nevertheless, this apparent robustness and abundance mask a structural dependence on natural resources, particularly sand, whose extraction is increasingly acknowledged as environmentally and economically unsustainable [5].

The scale at which concrete is produced places unprecedented pressure on natural aggregate resources [6,7,8,9]. Riverbeds, floodplains and coastal systems are being depleted at rates exceeding their natural replenishment, leading to habitat loss, altered sediment transport, reduced groundwater recharge and increased flood risk. Terrestrial aggregate quarrying is likewise associated with landscape degradation, noise and dust emissions, and long-term ecological disturbance [10]. In many regions, competition for high-quality fine aggregates has intensified to the point of triggering social conflicts and increasing public opposition. This has prompted calls for stricter regulation and more socially responsible extraction practices [11]. Together, these environmental and socioeconomic pressures underscore the urgent need to reduce the sector’s reliance on virgin aggregates and to transition towards more circular and resource-efficient construction practices [12].

One of the most widely explored strategies to mitigate this dependency is the use of construction and demolition waste (CDW) as a partial or full replacement for natural aggregates [13]. Over the past two decades, extensive research has characterised the mechanical and durability performance of recycled aggregates derived not only from concrete, but also from mixed masonry, ceramic and glass wastes, enabling their gradual incorporation into structural and non-structural concretes [14,15,16,17,18,19,20]. Several standards and design codes (national and international) now explicitly permit the use of such recycled aggregates under defined conditions, reflecting a growing level of industrial confidence [21,22]. However, this line of research, though highly valuable, primarily valorises residues generated within the construction cycle. Other waste streams with no direct link to concrete production, despite being abundant, low-value and environmentally problematic, remain comparatively underutilised.

Among these, end-of-life tyres (ELTs) constitute a persistent environmental challenge, as millions of tonnes accumulate annually due to their slow degradation, flammability and limited high-value recycling routes [23,24]. Incorporating ELT-derived shredded rubber as a partial sand replacement has therefore attracted growing interest as a means of simultaneously reducing natural aggregate consumption and diverting a problematic waste stream [25,26]. However, despite extensive research on mechanical behaviour, significant uncertainties remain regarding the durability and long-term performance of rubberised concrete, particularly under combined or multi-action exposure scenarios.

To address these gaps, the present study integrates a structured synthesis of existing evidence with an experimental programme designed to evaluate the mechanical behaviour, durability-related indicators and overall performance of rubberised concretes incorporating 5–50% ELT rubber (0/4 mm) as the selective replacement of a specific fine aggregate fraction. Beyond the assessment of conventional mechanical properties and durability indicators, particular emphasis is placed on quantifying material deterioration and the associated loss of mechanical capacity resulting from simultaneous exposure to freeze–thaw cycling and sulphate attack. This coupled action, representative of severe service conditions and which remains comparatively underexplored in the existing literature, provides a stringent framework for assessing the long-term performance of rubberised concretes.

## 2. Literature Review

The incorporation of end-of-life tyre (ELT) rubber into concrete can be approached through two main routes: the use of untreated rubber particles, directly obtained through mechanical shredding, and the use of treated rubber, in which the particle surface is subsequently modified to improve adhesion to the cementitious matrix [27,28,29]. Reported treatment strategies include alkaline washing, surface coatings, gamma irradiation, partial devulcanisation, thermal pre-treatment and the application of chemical coupling agents. These approaches are primarily intended to reduce the intrinsic hydrophobicity of rubber, refine the interfacial transition zone and mitigate the strength penalties typically associated with rubberised concretes [30,31,32,33,34,35,36,37,38,39,40,41,42,43,44,45].

Although surface treatments frequently result in improved mechanical performance and, in some cases, enhanced durability-related indicators, they also introduce additional processing steps, increased energy consumption and higher costs, together with practical constraints that limit their feasibility for large-scale industrial implementation. By contrast, untreated rubber (despite its well-documented drawbacks in terms of workability, strength reduction and increased porosity) remains the most widely investigated and arguably the most realistic pathway for implementation in conventional concrete production. Its simplicity of processing, compatibility with existing batching systems and absence of pre-treatment requirements make it more closely aligned with current construction practice. For these reasons, and in line with prevailing trends in the literature, the present review focuses exclusively on concrete mixtures incorporating untreated shredded rubber as aggregate replacement.

Over the past three decades, numerous studies have investigated the mechanical, physical and durability-related behaviour of rubberised concretes, giving rise to a broad but highly heterogeneous body of experimental evidence, as reflected in several state-of-the-art reviews [46,47,48,49,50,51,52,53,54,55,56,57,58,59,60]. Comparative analysis of this literature reveals substantial variability in testing protocols, exposure conditions and reported performance indicators, which hinders direct comparison across studies. Nevertheless, a consistent pattern can be identified: while mechanical performance has been extensively documented, durability-related properties or performance under aggressive or combined environmental actions are reported with far less consistency, and experimental investigations at structural or element scale remain largely absent.

**Figure 1:**
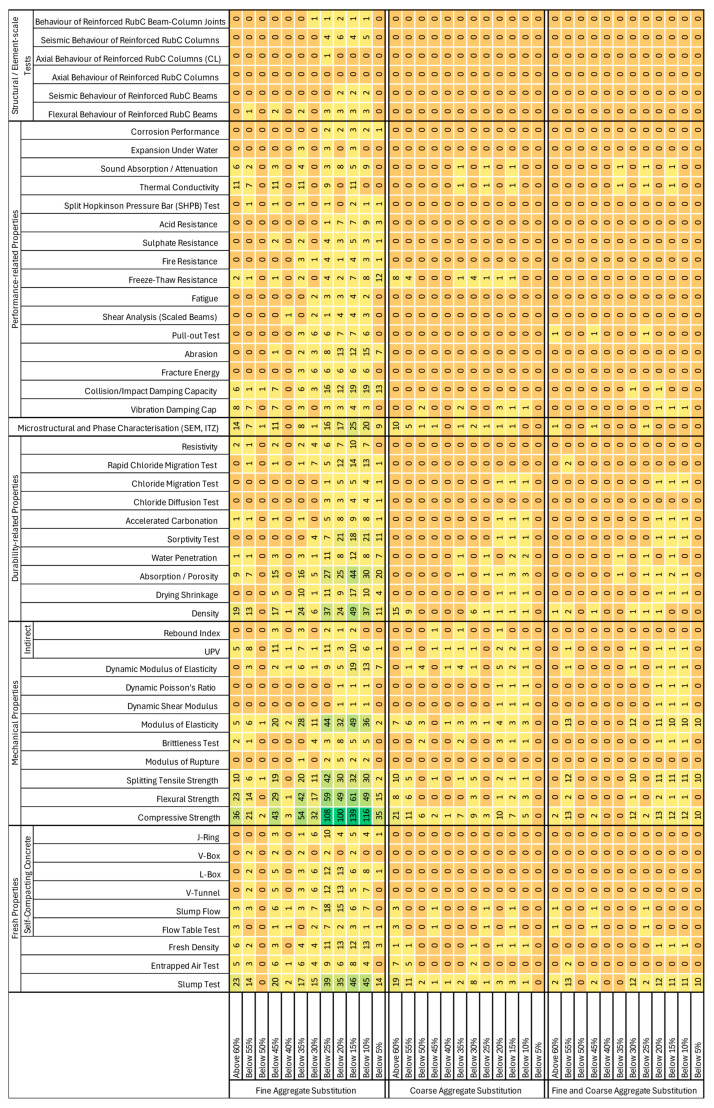
Heat-map of reported tests for rubberised concrete as a function of rubber replacement level and aggregate substitution strategy.

To structure and synthesise this fragmented evidence base, the present study undertook a targeted bibliographic screening aimed not merely at compiling existing data, but at systematically mapping how research effort has been distributed across rubber replacement levels and performance indicators. The frequency with which mechanical properties, durability-related parameters and exposure-specific tests have been reported was quantified as a function of rubber content, enabling the identification of both well-covered domains and persistent knowledge gaps. The resulting analysis, summarised in Figure 1, builds upon existing state-of-the-art review databases and an updated literature search, yielding a consolidated evidence base comprising more than 4500 experimental test results drawn from over 150 published studies [40,61,62,63,64,65,66,67,68,69,70,71,72,73,74,75,76,77,78,79,80,81,82,83,84,85,86,87,88,89,90,91,92,93,94,95,96,97,98,99,100,101,102,103,104,105,106,107,108,109,110,111,112,113,114,115,116,117,118,119,120,121,122,123,124,125,126,127,128,129,130,131,132,133,134,135,136,137,138,139,140,141,142,143,144,145,146,147,148,149,150,151,152,153,154,155,156,157,158,159,160,161,162,163,164,165,166,167,168,169,170,171,172,173,174,175,176,177,178,179,180,181,182,183,184,185,186,187,188,189].

To ensure conceptual clarity and comparability, the review was deliberately restricted to investigations employing recycled shredded rubber as the sole alternative constituent. Studies combining rubber with other materials, such as construction and demolition waste [190,191,192] or fibres [145,193,194,195,196], as well as those focusing on pervious concretes [197,198,199,200,201], were excluded in order to isolate the specific effects of rubber incorporation on concrete behaviour and performance.

### 2.1. Test Methods

As illustrated by the heat-map presented in Figure 1, research on rubberised concrete has expanded substantially; however, this expansion has been highly selective, resulting in a strongly uneven distribution of research effort across material properties, exposure conditions and aggregate replacement strategies. Rather than reflecting a balanced exploration of performance, the available literature reveals a pronounced concentration on a limited set of parameters and mixture configurations, leaving significant gaps in areas that are critical for assessing long-term behaviour and practical applicability.

**Figure 2:**
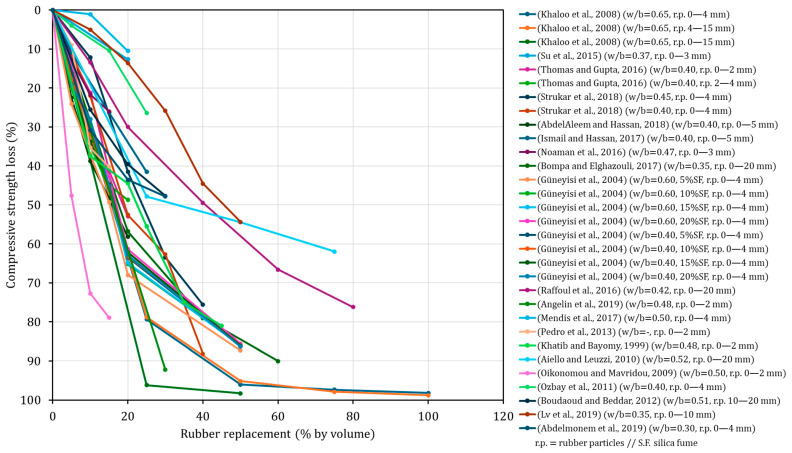
Compressive strength loss with increasing rubber content by volume [50,68,69,100,101,103,114,125,131,148,155,156,157,165,169,178,180,182,184,188].

The most intensively investigated domain is mechanical performance, with compressive strength, flexural strength, indirect tensile strength and elastic modulus clearly dominating the experimental landscape. This predominance reflects the central role of these parameters in material qualification and structural design, as well as their relative simplicity and high degree of standardisation compared to other performance indicators [202]. Across a wide range of mixture designs, rubber particle sizes, curing regimes and binder systems, most studies report abrupt reductions in mechanical properties, although these effects tend to be less pronounced at low replacement levels, typically below 5% to 10% rubber content. As shown in Figure 2, the synthesis of selected studies included in this literature review reveals a clear and systematic deterioration of mechanical properties, exemplified here by compressive strength (at 28 days), as rubber content increases, despite the substantial variability in specimen types, mixture proportions and experimental methodologies reported across the literature. This behaviour is commonly attributed to the poor mechanical compatibility between rubber particles and the cementitious matrix, which promotes the formation of a weak and stress-sensitive interfacial zone. However, despite the frequent invocation of this mechanism, only a limited number of studies have undertaken a detailed characterisation of the ITZ itself (Figure 1), and most interpretations rely on indirect evidence or qualitative observations derived from microstructural techniques such as SEM [107,141,168,174,185,188]. In addition, several authors have highlighted the pronounced mechanical contrast between conventional mineral aggregates and rubber particles, the latter exhibiting significantly lower stiffness and strength, and thus markedly lower rigidity than natural aggregates, which further compromises stress transfer within the composite [115,116,167]. The combined effects of poor stress transmission, increased microvoid formation and early crack initiation provide a coherent explanation for the observed mechanical degradation (Figure 1). While these trends are well established and repeatedly confirmed across the literature, their extensive documentation contrasts sharply with the limited attention devoted to other aspects governing service performance and long-term behaviour.

Fresh-state properties constitute a second major focus of the existing literature, as reflected in Figure 1. Conventional workability indicators such as slump and air content are frequently reported; however, in many studies these measurements are included primarily for descriptive purposes and do not form part of the core research objectives. In any case, a clear outcome emerging from the available data is the substantial dispersion of reported results. While a limited number of authors report no significant changes in workability following rubber incorporation [185], the majority observe negligible reductions at low replacement levels, followed by increasingly pronounced losses as rubber content increases [74,102,107,109,110,119,157,169,188]. At high replacement levels (typically beyond 50–60%) several studies report severely reduced workability, with mixtures becoming unworkable and prone to segregation [188]. These apparent contradictions are strongly influenced by differences in mixture design and by the use and dosage of chemical admixtures, which are not always reported in a consistent manner.

A significant subset of the literature has therefore concentrated on rubberised self-compacting concrete formulations, accounting for approximately 15% of the studies surveyed. These investigations typically report an extensive array of rheological characterisation tests, including slump flow, L-box, V-funnel and segregation resistance [85,103,117,130,148,165,167]. However, rather than demonstrating an intrinsic suitability of self-compacting systems for rubber incorporation, these studies are largely characterised by the systematic use of high contents of supplementary cementitious materials (SCMs), such as fly ash or silica fume [85,130,148,165,167], and in some cases fibre reinforcement, specifically introduced to stabilise the fresh mix and offset the adverse effects of rubber addition [103,117]. This formulation strategy enables the attainment of flowability and segregation resistance levels comparable to those of reference self-compacting concretes for rubber replacement levels of up to approximately 20%. For instance, Mallek et al. reported a reduction of only about 4% in slump flow with a 10% incorporation of crumb rubber [167], while Lv et al. observed a decrease of less than 6% in slump flow for rubber substitution levels as high as 20% [165]. Similarly, Güneyisi et al. reported no measurable change in slump flow diameter for rubber contents of up to 25%, albeit in mixtures where up to 60% of the cement content was replaced with fly ash [130]. These results highlight the effectiveness of SCM-rich formulations in preserving fresh-state performance; however, the considerable disparity in the selection and execution of rheological tests across studies hinders the establishment of clear and consistent comparative trends. Taking these limitations into account, it is nonetheless evident that, despite such formulation strategies, most studies ultimately report reductions in mechanical performance that are comparable in magnitude and trend to those previously described for non-self-compacting rubberised concretes.

Despite the comparatively extensive documentation of mechanical properties and fresh-state behaviour, durability-related investigations remain markedly less frequent and considerably more fragmented, as illustrated in Figure 1. Most studies addressing durability focus primarily on indirect physical indicators, such as water absorption, porosity accessible to water, sorptivity and electrical resistivity, which are typically assessed at early ages, most commonly at 28 days, under simplified laboratory conditions and for relatively narrow ranges of rubber content [69,76,85,98,102,104,108,119,124,126,132,168,181]. Across this body of work, and despite the diversity of test methods and experimental approaches presented, a broadly consistent trend can be identified. The incorporation of rubber is generally associated with increased porosity and water absorption, which is commonly reflected in greater carbonation depths compared to reference mixtures [77,102,132]. In parallel, rubberised concretes tend to exhibit a reduced resistance to ionic transport, as evidenced by lower electrical resistivity values or increased chloride diffusion coefficients [122,154,161,167]. These effects are most often attributed to the combined influence of weaker particle–matrix bonding and the lower stiffness and density of rubber particles [132].

From a durability standpoint, such trends are of particular concern, as increased permeability and reduced resistance to carbonation or chloride ingress are directly linked to an elevated risk of reinforcement corrosion, which remains one of the primary causes of durability loss and premature deterioration in reinforced concrete structures [203,204,205,206]. Nevertheless, among the more than 150 studies analysed, only two included experimental tests explicitly aimed at assessing corrosion processes in reinforced rubberised concrete [75,135]. Moreover, the findings of these studies remain difficult to generalise, as they were derived from relatively short exposure periods, limiting their relevance for long-term performance assessment [75,135].

Several exceptions to this general trend have been reported. In particular, for sorptivity and water absorption tests, a limited number of studies have observed reduced or unchanged values following rubber incorporation [95,161,180]. This behaviour is commonly attributed to the hydrophobic nature of rubber particles and their potential to hinder capillary water uptake, even in the presence of an overall increase in porosity [95,161,180]. However, it should be noted that these studies typically include additional mix design measures, such as the incorporation of fibres or SCMs, which are known to enhance concrete performance and may partially offset the adverse effects associated with rubber inclusion.

Similarly, some authors have reported an improved inhibitory effect on chloride ion transport with increasing rubber content, but only up to moderate replacement levels, typically below 15–20% [77,102,132,135,180]. At higher rubber contents, this effect may become neutral or even detrimental. In this case, the considerable variability and dispersion of the test methods and results reported in the literature must also be considered when interpreting these findings.

**Figure 3:**
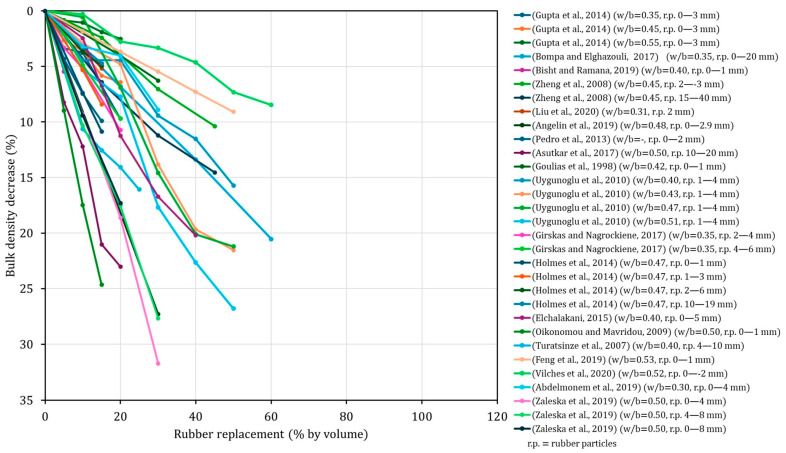
Bulk density decreases with increasing rubber content by volume [82,85,86,92,96,99,100,108,114,116,126,129,132,144,157,163,179,180,184].

In contrast to the scarcity of durability-related testing, bulk density is a property that has been extensively documented in the literature, with a large number of independent studies reporting comparable overall trends. Although the magnitude of the response is clearly dispersed, all published data consistently demonstrate a progressive reduction in bulk density with increasing rubber content. As illustrated in Figure 3, typical decreases of approximately 5–10% are already observed at moderate replacement levels (10–15% by volume), while reductions exceeding 15%, and in some cases approaching 30%, are frequently reported when rubber contents exceed 30%. This systematic behaviour directly reflects the substantially lower specific gravity of rubber particles compared to natural mineral aggregates (Figure 3). While such density reduction is occasionally highlighted as a potential advantage in terms of weight savings, impact mitigation or improved dynamic performance [207], it primarily reflects the lower intrinsic density of rubber compared to mineral aggregates. Although a decrease in bulk density does not inherently indicate increased porosity, it is frequently associated in practice with microstructural changes that adversely affect transport properties and long-term durability [208].

Beyond indirect durability indicators, investigations addressing specific deterioration mechanisms remain comparatively limited and are typically restricted to isolated exposure scenarios. Studies examining abrasion resistance, freeze–thaw resistance, sulphate attack, high-temperature exposure or fire performance are relatively few and rely on highly heterogeneous testing methodologies, exposure durations, rubber replacement levels and evaluation criteria [75,92,95,126,127,128,136,146,152,154,172,181,184,186]. This pronounced methodological dispersion, compounded by the wide range of mixture configurations investigated, significantly hampers direct comparison between studies and precludes the identification of consistent quantitative trends regarding the influence of rubber content or replacement strategy on a case-by-case basis, particularly under severe or prolonged environmental conditions. In many cases, assessment is largely confined to residual mechanical performance (compressive or flexural strength) measured after exposure, with limited attention paid to damage evolution, degradation kinetics or the governing physico-chemical mechanisms. As a result, the available evidence remains fragmented and largely qualitative, offering limited insight into the long-term behaviour of rubberised concrete under realistic service conditions or coupled deterioration processes.

In parallel, a limited number of studies have explored properties that are rarely considered in conventional concrete research but may be particularly relevant for rubberised materials, such as impact resistance [70,78,108,113,114,115,120,127,147,159,173,182,184], vibration damping [32,84,96,107,133,174], sound absorption [71,84,106,107,124,144,147,157] or thermal conductivity behaviour [71,84,92,105,107,112,147]. While these investigations often report promising functional advantages associated with rubber incorporation, they are typically based on narrow experimental programmes, sometimes non-standardised testing approaches and limited replication. Consequently, despite their potential relevance for niche applications, the existing data remain too sparse and heterogeneous to support generalisable conclusions across the literature.

A similar limitation applies to studies that have sought to advance understanding of rubberised concrete through the investigation of structural-scale elements. Among the more than 150 studies analysed in the present review, only a very small subset (14 works) has examined scaled structural components, such as beams subjected to flexure [148,170], columns under monotonic or cyclic axial loading [67,89], or beam and column elements assessed under seismic-type actions [88,90,137,138,139,140,162,175]. Even within this limited body of literature, experimental programmes differ substantially in terms of geometry, reinforcement detailing, rubber content, replacement strategy and loading protocols, which severely restricts meaningful comparison of results. Notably, only two studies have extended this approach to the combined loading behaviour of beam–column joints, despite their critical role in structural performance [61,118]. Consequently, although these pioneering studies provide valuable qualitative insights into the feasibility of rubberised concrete at the structural scale, the evidence base remains too limited and fragmented to support robust design-oriented recommendations.

Overall, the heat map analysis highlights that, despite the substantial growth in published research, experimental effort has been unevenly distributed, with a strong emphasis on mechanical properties and fresh-state behaviour, and comparatively limited, fragmented investigation of durability mechanisms, functional properties and structural performance. This imbalance, together with the pronounced heterogeneity of test methods, exposure conditions and mixture configurations, complicates meaningful comparison across studies and limits the development of generalisable performance trends. In particular, the limited attention devoted to coupled deterioration mechanisms, long-term behaviour and clearly defined aggregate replacement strategies reveals a critical gap in the current evidence base. Addressing these deficiencies requires more systematic, comparable and exposure-oriented research frameworks, particularly with respect to selective replacement approaches and their implications for durability under aggressive service conditions. These aspects are examined in the following section.

### 2.2. Crumb Rubber Replacement Strategy

A further critical insight provided by Figure 1 concerns aggregate replacement strategies. Studies combining the replacement of both fine and coarse aggregates are exceedingly scarce, largely due to the pronounced mechanical penalties typically associated with such approaches [101,125,155]. The simultaneous substitution of gravel and sand with rubber particles often leads to severe reductions in strength and stiffness, reflecting both the low rigidity of rubber and the difficulty of preserving an adequate granular skeleton when conventional aggregate gradations are substantially altered. Similarly, investigations focusing exclusively on coarse aggregate replacement remain limited to a very small number of studies, as the direct substitution of mineral gravel by rubber particles poses significant challenges in terms of particle size compatibility, load transfer and structural integrity.

As a result, the vast majority of published research has concentrated on the partial replacement of fine aggregates, which is generally perceived as a more manageable strategy from a mechanical standpoint. This imbalance, clearly illustrated in Figure 1, provides a comparative overview of the studies analysed, including the temporal distribution of publications, the ranges of rubber replacement levels and the specific substitution strategies adopted. While the predominance of fine aggregate replacement has enabled a large body of experimental work to be developed, it has also constrained the scope of the available evidence by limiting systematic exploration of alternative and potentially more efficient mix design strategies from both performance and waste valorisation perspectives.

A similarly conservative pattern emerges when replacement levels are considered. As shown in Figure 1, the literature is heavily concentrated within a relatively narrow range of rubber contents, most commonly below 30% by volume, with replacement levels exceeding 50–60% only sporadically investigated. This strong focus on moderate substitution levels reflects an implicit effort to limit mechanical degradation and preserve conventional performance benchmarks. However, while such levels may be suitable for exploratory studies or applications with limited structural demands, they provide only restricted insight into the feasibility, durability and long-term behaviour of rubberised concretes at higher rubber contents, which are arguably more relevant from a circular-economy and waste-management standpoint. Consequently, mixtures incorporating elevated replacement levels remain underrepresented, particularly with respect to durability-related indicators and performance under aggressive or coupled exposure conditions.

The patterns revealed by the heat-map (Figure 1) indicate that research on rubberised concrete has so far prioritised short-term material characterisation, primarily through mechanical and fresh-state testing, over systematic investigation of durability mechanisms and performance evolution. While the existing body of work supports the potential use of rubberised concretes in non-structural or semi-structural applications, where enhanced ductility, impact resistance or energy dissipation may partially offset reductions in strength, a robust understanding of long-term behaviour under realistic service conditions remains lacking. This limitation is particularly critical for applications in cold climates, coastal environments or sulphate-rich soils, where multiple deterioration mechanisms may act simultaneously.

Notably, an aspect that has received little attention in the existing literature is the selective replacement of a specific aggregate fraction within a well-graded reference concrete. Most studies replace either the entire fine aggregate fraction or a broad granulometric range, rather than adopting a multi-fraction reference gradation in which only a specific sand fraction is selectively substituted with rubber particles. This alternative approach offers the potential to preserve the overall granular skeleton and packing density of the mixture, thereby moderating mechanical degradation while still enabling meaningful levels of rubber incorporation.

These limitations provide the principal motivation for the experimental programme developed in the present study. In particular, the adoption of a selective aggregate replacement strategy, whereby end-of-life tyre rubber is used to substitute a specific sand fraction rather than the entire fine aggregate content, constitutes a deliberate methodological choice. This approach is intended to preserve the integrity of the granular skeleton and packing density of the reference mixture, thereby moderating mechanical degradation while maintaining meaningful levels of rubber incorporation. At the same time, the experimental framework moves beyond the predominantly short-term and single-exposure focus of much of the existing literature by explicitly addressing the combined action of freeze–thaw cycling and sulphate attack. This coupled deterioration scenario, representative of aggressive service environments, remains comparatively underexplored despite its practical relevance in cold and chemically aggressive conditions. By integrating mechanical characterisation, durability-related indicators and performance-based assessment under interacting environmental actions, the study seeks to provide a more comprehensive and mechanistically informed evaluation of rubberised concrete behaviour.

Accordingly, the key gaps identified in the literature review are directly addressed in the mix design and experimental programme described in Section 3, notably through the selective replacement of a specific sand fraction and the deliberate assessment under combined freeze–thaw and sulphate attack, a coupled exposure condition that remains comparatively underexplored in the existing literature.

## 3. Materials and Methods

### 3.1. Materials

A reference concrete mix was first designed to achieve a compressive strength above 45 MPa at 28 days, representative of structural concrete commonly used in infrastructure applications. A relatively high water–cement ratio (w/c = 0.55) was adopted, in accordance with the limits established in the Spanish Structural Code [21], combined with a superplasticiser (SP-Sika^®^ ViscoCrete^®^-5970) (Valencia, Spain) to ensure adequate workability. CEM I 42.5 R cement supplied by Lafarge (Madrid, Spain) was used. The aggregate skeleton consisted of:

- A siliceous gravel, fraction 4/12 mm (SG 4/12));
- A siliceous sand, fraction 0/4 mm (SS 0/4);
- A siliceous fine sand, fraction 0/2 mm (SS 0/2);
- A limestone filler (LF) used to improve packing density and the overall fineness of the aggregate blend.

The combination of these fractions was selected to obtain a continuous and well-graded particle size distribution, promoting adequate compaction and reduced porosity in the reference concrete, while enabling the straightforward substitution of the 0/4 mm sand with rubber.

**Figure 4:**
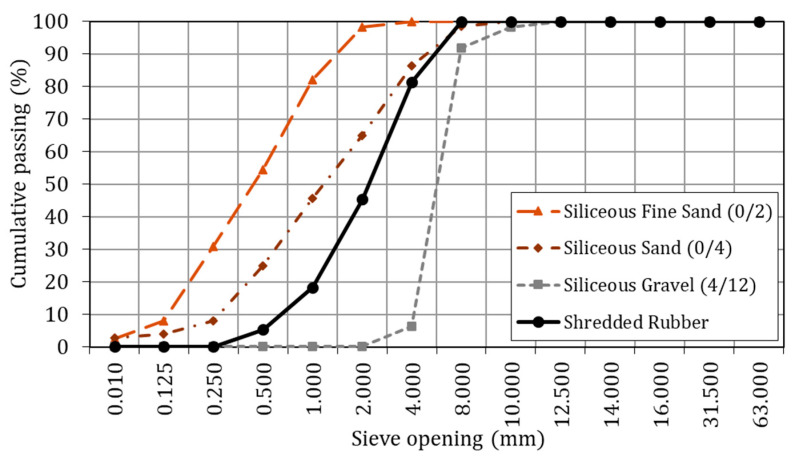
Shredded rubber used and grading curves of sand and rubber particles.

The rubber used in this study was supplied by Agrogardening (Cuenca, Spain) as a commercial product typically employed in gardening and drainage applications, and was produced from 100% shredded end-of-life tyres. The material presented a specific gravity of 0.85, a bulk unit weight of 0.63 kg/dm^3^, and a fineness modulus of 3.05, reflecting its lightweight nature. Prior to mix design, the material was sieved to characterise its particle size distribution and to compare it with that of the siliceous sands used in the reference mixture. As shown in Figure 4, the rubber exhibits a particle size distribution broadly comparable to that of the 0/4 mm siliceous sand, while presenting a markedly lower proportion of fine particles. This grading clearly differs from that of the 0/2 mm sand and more closely resembles the coarser portion of the fine aggregate fraction. On this basis, the rubber was deemed more suitable for the selective replacement of the coarser sand fraction within the 0/4 mm range, rather than for the substitution of the entire fine aggregate content.

The mix proportions of the reference concrete (CR-00) per cubic meter are indicated in Table 1:

Seven additional rubberised concrete mixtures (CR-05 to CR-50) were produced by replacing 5, 10, 15, 20, 30, 40 and 50% of the 0/4 mm sand by mass with recycled rubber granulate, while keeping all other constituents constant.

After casting, all specimens manufactured within the present experimental programme were kept in their moulds for 24 h and protected with plastic sheets to minimise early-age moisture loss and prevent plastic shrinkage cracking. Following demoulding, the specimens were carefully labelled and transferred to a controlled curing chamber maintained at 20 ± 2 °C and a relative humidity above 95%. This curing regime was applied consistently to all mixtures and specimen types until the designated testing age, which was 28 days.

### 3.2. Test Methods

All tests were performed at 28 days. Compressive strength was determined on 100 mm cubes in accordance with EN 12390-3:2020 [209], while flexural strength was measured on 100 × 100 × 400 mm prisms following EN 12390-5:2020 [210]. The rebound index was obtained using a Schmidt Hammer (digital model supplied by Mecánica Científica, Madrid, Spain) in accordance with EN 12504-2:2022 [211] on the surface of 100 × 100 × 400 mm prisms; ultrasonic pulse velocity (UPV) measurements were carried out on the same specimens following EN 12504-4:2022 [212]. Bulk density and water-accessible porosity were determined in accordance with UNE 83980:2014 [213] on cylindrical specimens with a diameter of 100 mm and a height of 50 mm. Carbonation resistance was evaluated on 40 × 40 × 160 mm prisms exposed to an accelerated carbonation environment, with CO_2_ concentration, temperature and relative humidity controlled according to UNE 83993-2:2013 [214] (CO_2_ = 3.0 ± 0.5%; T = 23 ± 2 °C; RH = 65 ± 5%). The chloride diffusion coefficient was determined using an accelerated migration method in accordance with EN 12390-18:2021 [215]. Electrical resistivity was measured on 40 × 40 × 160 mm prisms following the direct method described in EN 12390-19:2023 [216].

To evaluate performance under highly aggressive conditions, an additional test combining freeze–thaw cycling and sulphate attack was performed. This combined exposure scenario was specifically selected in view of the limited attention devoted in the literature to coupled deterioration mechanisms in rubberised concrete, with most existing studies addressing these actions in isolation. As no standardised test procedure is currently available for the simultaneous assessment of freeze–thaw and sulphate exposure, the experimental protocol adopted in this study was deliberately defined by integrating and adapting relevant provisions from existing standards addressing each mechanism separately.

Four 100 mm cubes were prepared for each mixture. Under reference conditions, two cubes were stored in tap water at 23 ± 2 °C for 270 days. Under aggressive conditions, the remaining two cubes were immersed in a 10% MgSO_4_ solution (0.35 M), prepared in accordance with ASTM C1012:2004 [217], and placed in a climatic chamber programmed to apply daily temperature cycles between +20 °C and −20 °C, following the temperature regime specified in CEN/TS 12390-9:2006 [218]. Each cycle lasted 24 h, and the combined exposure was maintained for approximately 270 days (nine months).

After the exposure period, all specimens were visually inspected to identify surface damage, cracking or material loss, and subsequently tested in compression in accordance with EN 12390-3:2020 [209]. The residual compressive strength obtained under aggressive conditions was subsequently compared with that of the water-cured reference specimens.

## 4. Results and Discussion

### 4.1. Mechanical Properties

#### 4.1.1. Compressive Strength

**Figure 5:**
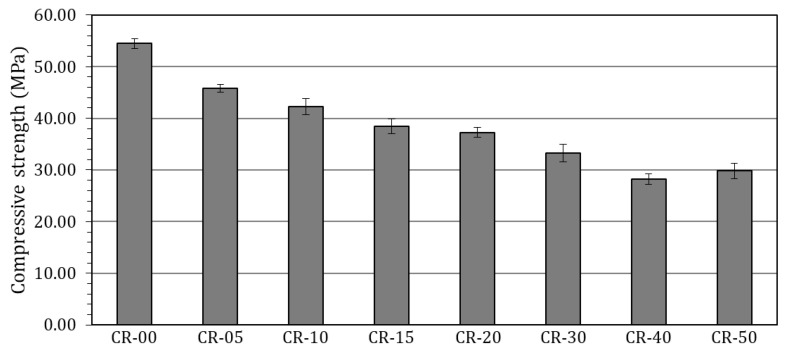
Compressive strength results.

Figure 5 illustrates the evolution of compressive strength at 28 days as a function of rubber replacement level, expressed as the proportion of shredded ELT rubber incorporated relative to the selected sand fraction.

The reference concrete (CR-00) achieved a compressive strength of 54 MPa at 28 days. The incorporation of ELT rubber as a selective replacement of the 0/4 mm sand fraction resulted in a systematic reduction in compressive strength with increasing rubber content (Figure 5). A replacement level of 5% (CR-05) led to a decrease to around 46 MPa, corresponding to a strength loss of approximately 15% relative to the control mix. Further increases to 10%, 15% and 20% replacement (CR-10, CR-15 and CR-20) yielded compressive strengths of about 42 MPa, 38 MPa and 37 MPa, respectively, corresponding to strength losses ranging from 22% to 30%. Beyond this threshold, the detrimental effect of rubber incorporation became more pronounced, with compressive strengths decreasing to approximately 33 MPa for CR-30, 28 MPa for CR-40 and 30 MPa for CR-50, corresponding to strength losses ranging from 40% to 48%.

When these results are interpreted in the context of the broader literature (Figure 2), it is important to clarify that the rubber replacement levels reported in the present study are not directly equivalent to those commonly adopted in previous works. In the present study, rubber was used as a selective replacement of a specific sand fraction, such that the highest nominal replacement level of 50% corresponds to approximately 25% of the total fine aggregate content. This distinction is essential to ensure a meaningful comparison with studies in which rubber replaces the entire fine aggregate fraction.

Taking this into account, the strength loss observed in the present study can be considered comparatively moderate, particularly at low to moderate effective replacement levels. For studies employing rubber particles within a similar size range (0–4 mm), compressive strength losses at an effective fine aggregate replacement of around 25% (equivalent to 50% in the present study) are frequently reported to be on the order of 65–80% [131,155], whereas the corresponding reduction observed here is approximately 45%. Likewise, at lower replacement levels, an effective substitution of about 10% of total fine aggregates (corresponding to 20% in the present study) typically results in strength losses of 35–45% [114,131,155] in the literature for comparable rubber particle size distributions, while the present results show reductions of less than 30%. These comparisons indicate that the selective substitution of a well-defined sand fraction, rather than complete replacement of the fine aggregate content, can substantially mitigate the severity of compressive strength degradation. This approach appears to preserve a greater proportion of the load-bearing mineral skeleton while still enabling meaningful levels of waste rubber valorisation, thereby moderating the otherwise abrupt strength decay widely reported for rubberised concretes.

These observations do not imply a change in the fundamental mechanisms governing strength degradation in rubberised concretes, but rather a moderation of their impact as a result of the adopted replacement strategy. The progressive loss of compressive strength observed remains consistent with the mechanisms widely reported in the literature, including the substantially lower stiffness and strength of rubber compared to mineral aggregates, the development of a weak interfacial transition zone (ITZ) between rubber particles and the cementitious matrix, and the associated increase in porosity and reduction in packing density.

#### 4.1.2. Flexural Behaviour

**Figure 6:**
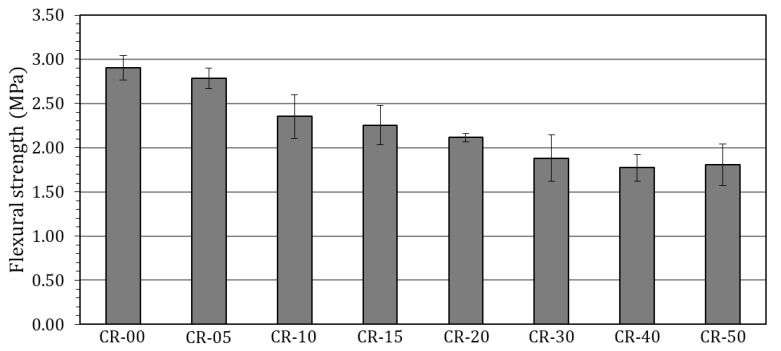
Flexural strength results.

Figure 6 shows the flexural strength results at 28 days, providing complementary insight into the mechanical response of rubberised concretes under bending.

As can be observed, the incorporation of ELT rubber leads to a progressive reduction in flexural strength with increasing replacement level, with the magnitude of this reduction remaining relatively moderate at low to moderate contents when compared with values commonly reported in the literature. For replacement levels up to 20%, flexural strength values remain at reasonably high levels, corresponding to an average reduction of approximately 25% relative to the reference concrete. Only a limited number of studies have reported comparable flexural strength losses at similar effective replacement levels [76,92], whereas the majority of published works indicate substantially higher reductions, frequently exceeding 40% [119] and in some cases approaching or surpassing 50% [180].

At higher replacement levels (30–50%), flexural strength decreases more markedly, with losses ranging from approximately 30% to 40%, reflecting the cumulative influence of increased porosity, reduced packing density and the diminishing contribution of the stiff mineral aggregate skeleton. This behaviour is consistent with the trends observed in compressive strength tests. Nevertheless, for comparable effective replacement levels and similar rubber particle size ranges, the flexural strength loss recorded in the present study remains significantly less abrupt than that typically reported in the literature. For example, at similar substitution levels, Záleská et al. reported flexural strength losses exceeding 78% for 30% substitution [92], with comparable reductions also documented by Angelin et al. (75% for 30% substitution) and Elchalakani (72% for 40% substitution) [108,168]. This further supports the premise that the selective substitution of a specific sand fraction moderates the deterioration of bending performance, although the beneficial effects associated with increased deformability are no longer sufficient to compensate for the overall mechanical penalties at high rubber contents.

#### 4.1.3. Non-Destructive Indicators: Rebound Index and UPV

Table 2 summarises the rebound index and ultrasonic pulse velocity (UPV) values obtained for all mixtures at 28 days. These non-destructive indicators provide complementary information on surface hardness and internal material integrity, respectively, and allow trends associated with increasing rubber content to be assessed alongside the mechanical and durability-related results discussed previously.

As shown in Table 2, the rebound index generally decreases with increasing rubber content, in agreement with the reductions observed in compressive and flexural strength. The reference concrete (CR-00) exhibits the highest rebound value, whereas mixtures incorporating higher rubber contents, particularly beyond 30%, show a clear deterioration in rebound response. Nevertheless, the evolution of the rebound index is less systematic than that of strength, reflecting the well-known sensitivity of this test to surface conditions and local heterogeneities. In contrast, ultrasonic pulse velocity exhibits a clearer and more systematic decline with increasing rubber content, indicating a progressive degradation of the internal structure. The reference concrete presents UPV values of approximately 4350 m/s, which, according to the quality classification proposed in [212], corresponds to concrete of good to excellent quality. Mixtures incorporating low rubber contents (up to 5%) remain within this quality range [212], while concretes incorporating 10–20% rubber exhibit UPV values between approximately 4000 and 4100 m/s, corresponding to a reduction of about 5–10% relative to the control mix and still classified as good quality concrete [212]. At higher replacement levels (30–50%), UPV values decrease to the range of approximately 3650–3850 m/s, representing a reduction of roughly 15% compared to the reference concrete and corresponding to acceptable quality concrete according to the same classification [212]. This transition reflects a clear increase in internal discontinuities, reduced stiffness and a less compact microstructure, in line with the results discussed previously. Similar UPV reductions with increasing rubber content have been widely reported in the literature [32,85,91], and are mainly attributed to the significantly higher absorption of acoustic vibrations by rubber particles compared to mineral aggregates, which, together with the increased porosity and weaker interfacial transition zones previously discussed, governs wave attenuation and velocity reduction in rubberised concretes [32,155,219].

Overall, the combined rebound and UPV results confirm that rubber incorporation progressively alters both surface hardness and internal integrity, with UPV proving to be a more reliable indicator of internal microstructural deterioration. Importantly, despite the observed reductions, concretes incorporating moderate rubber contents still fall within quality ranges considered acceptable or good according to established UPV-based classifications [212], further supporting the effectiveness of the selective sand-fraction replacement strategy adopted in this study.

### 4.2. Transport Properties and Durability Indicators

#### 4.2.1. Density and Water-Accessible Porosity

**Figure 7:**
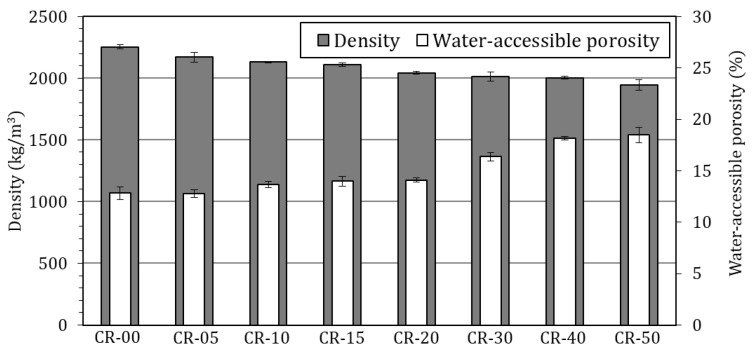
Density and water-accessible porosity.

Figure 7 shows the bulk density and water-accessible porosity of all mixtures. Both parameters are commonly used as indirect durability indicators, since they govern transport properties and strongly affect the susceptibility of concrete to the ingress of aggressive substances [220].

As expected, bulk density decreases progressively with increasing rubber incorporation, reflecting the substantially lower specific gravity of ELT rubber compared to natural aggregates. For low to moderate replacement levels (≤20%), the reduction in density remains relatively limited, not exceeding 10% with respect to the reference concrete. At higher replacement levels, the decrease becomes more pronounced, although still moderate in absolute terms, reaching reductions of 10.6%, 11.2% and 13.7% for 30%, 40% and 50% rubber incorporation, respectively. This behaviour is consistent with the trends widely reported in the literature for rubberised concretes (Figure 3) and confirms that density reduction is an inherent consequence of rubber incorporation. Such reductions are often interpreted both as an indirect indicator of increased void content and, in some cases, as a potential advantage for lightweight or impact-mitigating applications [207].

On the other hand, water-accessible porosity exhibits a complementary, but more durability-relevant, response. Porosity values increase with rubber content, with a noticeable change in slope beyond the 20–30% replacement range. For low to moderate rubber levels (≤20%), porosity remains relatively close to that of the reference concrete, ranging from approximately 12% to 14%. This suggests that the adopted grading strategy, based on the selective replacement of a specific sand fraction while retaining the 0/2 sand and filler, was effective in maintaining acceptable packing density and limiting pore connectivity. According to durability classifications commonly reported in the literature, porosity values within this range are typically associated with average to good durability performance for concrete [221,222]. This behaviour contrasts with many published studies in which rubber replaces the entire fine aggregate fraction, often resulting in much sharper increases in accessible porosity even at relatively low replacement levels; for instance, Bisht and Ramana reported porosity increases exceeding 25% for rubber contents below 5% [98].

At higher rubber contents (≥30%), water-accessible porosity increases more markedly, indicating the development of a more connected pore network. This behaviour can be attributed to the reduced ability of rubber particles to properly adhere to the cement paste, leading to weaker particle–matrix interactions and promoting the formation of interfacial voids and microgaps. The observed porosity increase, corresponding to relative increments of approximately 30% to 45% compared to the reference concrete, is consistent with trends reported in the literature and supports the interpretation of progressive microstructural degradation at high replacement levels [85,122,126,168]. According to reference ranges commonly used to assess durability potential, the porosity values obtained for mixtures with ≥30% rubber substitution are typically associated with lower durability performance, implying increased susceptibility to the ingress of aggressive agents [221,222].

Overall, the combined density and water-accessible porosity results confirm that the selective sand-fraction replacement strategy adopted in this study effectively moderates the deterioration of transport-related properties at low to moderate rubber contents, while higher replacement levels lead to microstructural changes that may significantly compromise long-term durability.

#### 4.2.2. Carbonation Resistance

**Figure 8:**
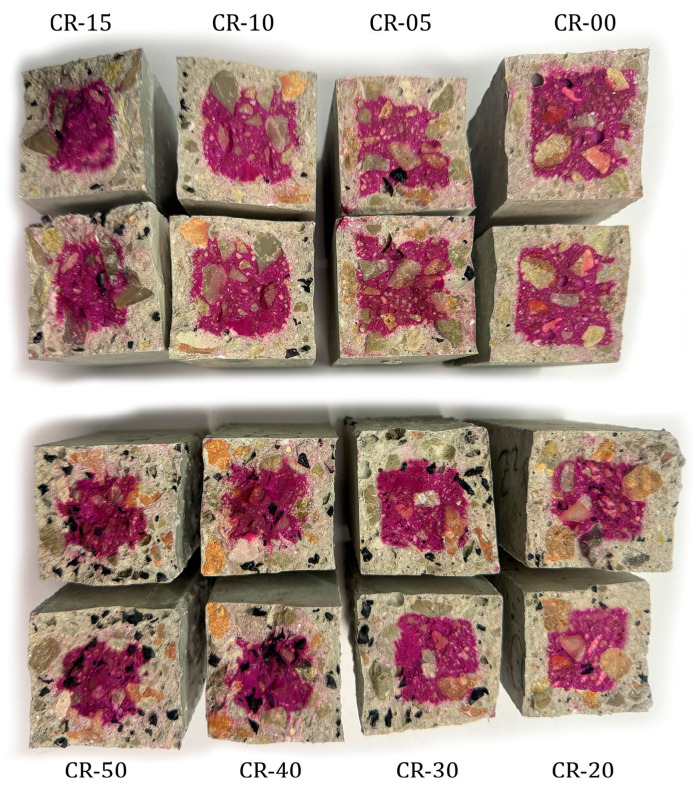
Carbonation analysis.

Carbonation is one of the primary mechanisms leading to reinforcement corrosion in reinforced concrete structures, as it leads to a reduction in the alkalinity of the cementitious matrix and the depassivation of the steel [223]. Figure 8 presents the results of the carbonation tests performed after the exposure period, illustrating the carbonation fronts observed in the different mixtures. In addition, Table 3 summarises the average carbonation depths measured for each mixture, allowing a quantitative comparison of the effect of increasing rubber content on carbonation resistance.

The reference concrete exhibited a relatively shallow carbonation depth (9.0 mm), reflecting its dense microstructure and low connectivity of the pore network. The incorporation of limited amounts of ELT rubber led to only modest increases in carbonation depth. Concretes with rubber replacement levels up to 20% showed carbonation depths between 9.75 and 11.25 mm, representing increases ranging from 10% to 25% relative to the reference mixture. These variations remain relatively limited and suggest that, at low to moderate replacement levels, the adopted selective replacement and grading strategy is also effective in preserving a sufficiently compact cementitious matrix, thereby restricting CO_2_ ingress.

A markedly different behaviour was observed for higher rubber contents. Beyond the 20–30% replacement threshold, carbonation depth increased more sharply, reaching values of 13.75, 14.25 and 15.25 mm for the 30%, 40% and 50% rubber mixtures, respectively. This change in slope mirrors the trends previously identified in porosity and indicates a significant increase in pore connectivity and gas transport capacity at high rubber contents.

The influence of rubber incorporation on carbonation depth has been comparatively less investigated than other durability-related properties. Among the more than 150 studies analysed in the literature review presented earlier, only five report carbonation data. Despite this limited body of evidence, the trends identified are largely consistent with the results obtained in this study. In most cases, rubberised concretes with low replacement levels exhibit carbonation depths only slightly higher than those of the reference mixtures, whereas significantly greater carbonation penetration or accelerated carbonation rates are reported at higher rubber contents. For instance, Gupta et al. observed that for a rubber content of 15%, the increase in carbonation depth remained below 15% compared to the control concrete [132]. However, once the rubber content exceeded 20%, the carbonation depth became approximately four times greater than that of the reference mixture [132]. Comparable increases at higher replacement levels were also reported by Bravo and Brito [102].

A different trend was observed in some studies at low replacement levels. Thomas et al. [77] and Vilches et al. [86] reported reductions in carbonation depth of up to 10% for rubber contents below 12.5% and 30%, respectively. These authors suggested that replacing natural aggregates with rubber particles of similar grading may locally reduce carbonation susceptibility, potentially due to the hydrophobic nature of rubber and its ability to disrupt capillary water continuity, thereby limiting the progression of the carbonation reaction [77]. Nevertheless, for higher replacement levels, both studies reported a clear increase in carbonation depth, in agreement with the trends observed in the present work and with the concomitant increases in porosity and transport properties [77,86].

Mallek et al. were the only authors to report a systematic reduction in carbonation depth across all the mixtures analysed, with decreases of approximately 10% for concretes incorporating up to 15% rubber as a replacement of the 0–8 mm aggregate fraction [167]. However, it should be noted that this study employed accelerated carbonation conditions with CO_2_ concentrations of up to 15%, which are significantly higher than the 3–5% CO_2_ levels typically used in the literature and recommended by most standards. While several studies indicate that accelerated carbonation tests conducted at around 3% CO_2_ produce trends comparable to those observed under long-term natural carbonation conditions [224], exposure levels exceeding 10% CO_2_ may induce pore structure modifications that differ from those occurring under natural exposure. Consequently, carbonation results obtained under such extreme conditions should be interpreted with caution [225].

From a practical perspective, the results suggest that rubberised concretes incorporating up to 15–20% ELT rubber may retain acceptable resistance to carbonation for applications in non-aggressive exposure classes, particularly when used in non-reinforced or lightly reinforced elements. In contrast, mixtures with rubber contents of 30% or higher exhibit significantly increased susceptibility to carbonation and would require additional protective measures or mix design modifications if intended for reinforced concrete applications.

#### 4.2.3. Chloride Diffusion Coefficient

**Figure 9:**
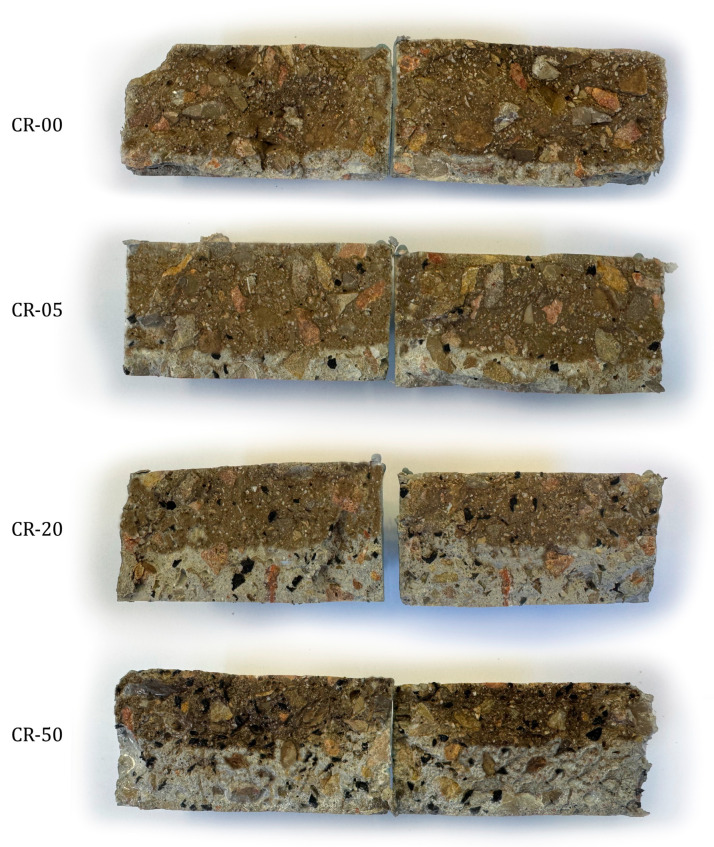
Chloride ion penetration.

**Figure 10:**
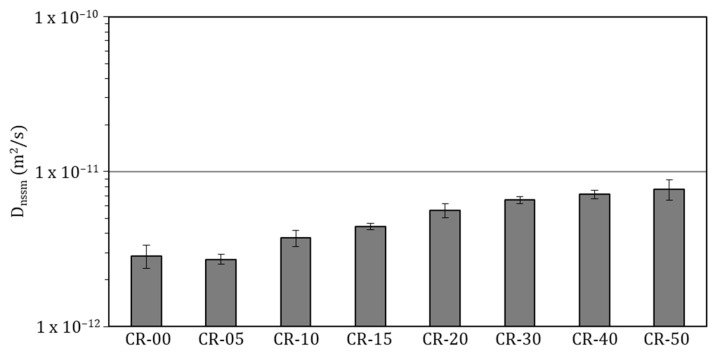
Chloride diffusion coefficient results.

The depassivation of reinforcing steel may also occur because of chloride ingress through the porous cementitious matrix; therefore, assessing the resistance of concrete to chloride ion transport is of fundamental importance for durability evaluation. Figure 9 illustrates the chloride penetration fronts revealed after the migration test, while Figure 10 presents the results obtained from the accelerated chloride migration test, which was used to quantify the resistance of the different mixtures to chloride penetration. The resulting migration coefficients enable a comparative assessment of the influence of rubber incorporation on chloride transport behaviour and complement the trends observed in electrical resistivity and water-accessible porosity measurements.

The results obtained reveal a progressive increase in the non-steady-state chloride migration coefficient with increasing rubber content, in agreement with the trends previously identified for other transport-related and durability indicators. For the reference concrete and mixtures with low rubber contents (CR-05 and CR-10), the $D_{nssm}$ values remain very close to those of the conventional concrete (approximately 2.8 × 10^−12^ m^2^/s), indicating no significant deterioration in resistance to chloride ingress. According to commonly reported durability classifications in the literature, chloride migration coefficients within this range can be associated with concretes exhibiting high durability performance [221,222]. This behaviour is consistent with the limited variations observed in water-accessible porosity or carbonation resistance at these replacement levels, and suggests that, up to approximately 10–15% rubber incorporation, the selective replacement of a specific sand fraction proposed in this study effectively preserves a relatively dense cementitious matrix with a weakly connected pore network.

From rubber contents of 15–20% onwards, a more pronounced increase in the migration coefficient is observed, with a clear change in slope for mixtures CR-30, CR-40 and CR-50, which reached values of 6.55, 7.15 and 7.68 × 10^−12^ m^2^/s, respectively. These values are generally associated in the literature with concretes of average to low durability. This tendency closely matches that identified in the previous test. Higher rubber contents lead to significant modifications of the concrete microstructure, characterised by increased connected porosity, greater heterogeneity of the cementitious matrix and the presence of weaker interfacial transition zones surrounding rubber particles. These features collectively facilitate ionic transport under an applied electrical field.

Among the studies reporting comparable chloride migration tests, Bravo and Brito are among the few authors to present results obtained using a similar methodology, reporting $D_{nssm}$ values ranging between 10 and 16 × 10^−12^ m^2^/s for rubber replacement levels between 5% and 15% [102]. These values indicate a more pronounced deterioration in chloride resistance compared with the results obtained in the present study. In addition, those authors observed that replacing coarse aggregates led to a more severe degradation of transport properties, whereas mixtures in which only fine aggregates were replaced exhibited improved performance. Nevertheless, even in the latter case, the reported chloride resistance remained lower than that achieved in this study, highlighting the effectiveness of selectively replacing a specific sand fraction rather than the entire fine aggregate content.

#### 4.2.4. Electrical Resistivity

**Figure 11:**
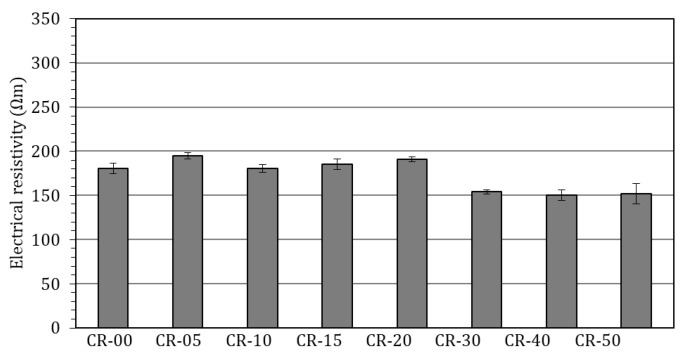
Electrical resistivity results.

Electrical resistivity represents an important durability-related property, as it reflects the ability of the cementitious matrix to transport electric charges through its pore solution. Since ionic transport is closely associated with pore structure, moisture content and pore connectivity, electrical resistivity is widely used as an indirect indicator of transport properties and overall durability performance. Resistivity measurements are frequently correlated with susceptibility to reinforcement corrosion, chloride ingress and other degradation mechanisms governed by mass transport processes. Figure 11 summarises the electrical resistivity values obtained for all the mixtures analysed.

The reference concrete (CR-00) and the mixtures incorporating rubber contents up to 20% (CR-05, CR-10, CR-15 and CR-20) exhibited resistivity values ranging from 180 to 200 Ωm. These values fall within the range typically associated with average durability performance [221,222]. Notably, no systematic decrease in resistivity was observed within this range of rubber contents, suggesting that the selective replacement strategy adopted effectively preserves the continuity of the cementitious matrix at low to moderate substitution levels. A more pronounced reduction in resistivity was observed for higher rubber contents. From CR-30 onwards, resistivity values decreased to approximately 150 Ωm for CR-30 and remained within the range of 140–150 Ωm for CR-40 and CR-50. Although these values are still classified within the lower bound of the “average” durability category according to the literature [221,222], they indicate a clear trend towards poorer transport resistance and a progressive approach to the threshold below which durability is commonly considered unsatisfactory (often reported around 100 Ωm). This behaviour reflects the increasing influence of rubber incorporation on pore connectivity and ionic mobility.

Electrical resistivity is another durability-related parameter that has received relatively limited attention in the literature on rubberised concrete. Among the studies analysed in the present review, only four report resistivity measurements, and all of them focus exclusively on surface resistivity, typically assessed using the Wenner four-point probe method [91,123,146,181]. Despite differences in testing procedures, these studies consistently report trends comparable to those observed in the present work, with electrical resistivity remaining relatively unchanged for rubber contents below approximately 15–20% [91,123,146,181].

Gheni et al. are the only authors to have investigated bulk electrical resistivity in rubberised concretes [123]. Their results indicated a slight increase in resistivity for replacement levels up to 20%, followed by an abrupt decrease at higher rubber contents, with reported losses exceeding 50% relative to the reference concrete [123]. This behaviour closely mirrors the change in slope identified in the present study and further supports the existence of a threshold rubber content beyond which microstructural degradation and enhanced ionic transport become dominant.

The reduction in electrical resistivity observed at high rubber contents can be attributed to the combined effects of increased water-accessible porosity, enhanced moisture retention and the development of more continuous transport pathways within the cementitious matrix. This behaviour is consistent with the trends identified for porosity, carbonation and chloride-related indicators, confirming that microstructural degradation becomes dominant beyond the 20–30% replacement range. Overall, the resistivity results corroborate the findings derived from the other durability-related parameters investigated in this study: while moderate rubber contents (≤20%) do not critically compromise transport properties, higher replacement levels lead to a significant deterioration of the microstructure and may adversely affect long-term durability, particularly under chloride exposure or other aggressive environmental conditions.

### 4.3. Performance Under Combined Freeze–Thaw and Sulphate Attack

**Figure 12:**
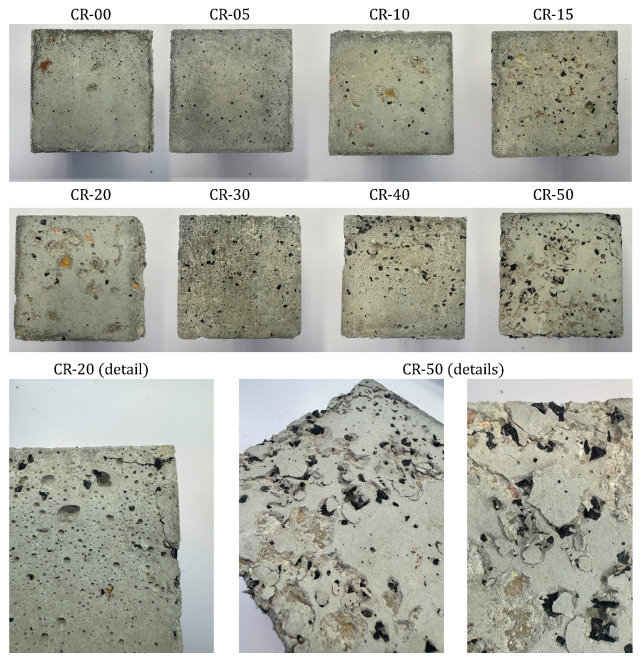
Visual inspection of test specimens after 270 freeze–thaw daily cycles in 10% MgSO_4_.

**Figure 13:**
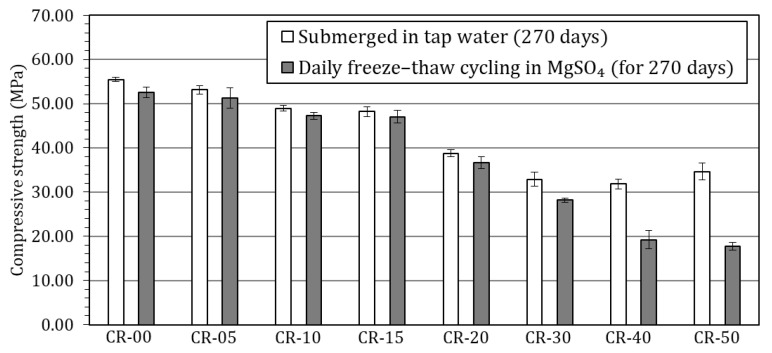
Compressive strength of reference and exposed specimens after 270 days under combined freeze–thaw cycling in MgSO_4_ solution and water storage.

The most distinctive aspect of this study is the evaluation of concrete performance under severe coupled environmental actions, combining freeze–thaw cycling with sulphate attack. After 270 daily cycles in a 10% MgSO_4_ solution, visual inspection revealed progressive surface deterioration that intensified with increasing rubber content (Figure 12). Figure 13 presents the compressive strength results of the exposed specimens, together with those of the corresponding reference specimens cured in tap water for 270 days.

Visual inspection after exposure to the combined freeze–thaw cycling and sulphate attack revealed damage patterns strongly dependent on rubber content. For the reference concrete and mixtures incorporating up to 20% rubber, surface deterioration was limited to slight surface roughening. The cementitious matrix remained largely cohesive, and no significant spalling or material loss was observed. In contrast, mixtures containing 30% or more rubber exhibited substantially more severe surface degradation, characterised by pronounced scaling, local disintegration with loss of material, fissuring, and areas with loose paste and exposed aggregates. This deterioration became particularly severe in the mixtures with 40% and 50% rubber, indicating a marked loss of structural integrity under the applied aggressive conditions.

These visual observations are consistent with the compressive strength results presented in Figure 13. For the reference concrete and mixtures incorporating up to 20% rubber, the compressive strength after exposure to the combined freeze–thaw cycling and sulphate attack remained close to that of the corresponding specimens stored in tap water. Strength losses were limited to 5.0% in the reference concrete and to 3.5%, 3.5%, 2.4% and 5.3% for CR-05, CR-10, CR-15 and CR-20, respectively, indicating a relatively minor degradation under the applied aggressive conditions. In contrast, a markedly different behaviour was observed for higher rubber contents. Mixtures CR-30, CR-40 and CR-50 exhibited much more pronounced strength losses, reaching 14.2%, 39.6% and 48.8%, respectively. These abrupt reductions reflect the severe internal damage previously identified through visual inspection and confirm that, beyond a critical rubber replacement level, the beneficial effects associated with rubber incorporation are outweighed by microstructural deterioration.

The observed degradation must be interpreted as the result of a synergistic interaction between sulphate attack and freeze–thaw cycling. Sulphate attack primarily affects the cement paste through the formation of expansive reaction products such as ettringite and gypsum, generating internal tensile stresses, microcracking and progressive matrix softening [226,227,228]. Under freeze–thaw exposure alone, deterioration is mainly physical, driven by hydraulic and crystallisation pressures associated with ice formation within the pore network. However, under combined exposure, sulphate-induced microcracking and decalcification act as a preconditioning stage that increases pore connectivity and degree of saturation, rendering the matrix significantly more vulnerable to frost action. Subsequent freezing cycles then accelerate crack propagation and crack coalescence within the already weakened microstructure, leading to rapid stiffness loss and surface scaling. Therefore, the deterioration observed under combined conditions cannot be interpreted as a simple additive effect of two independent mechanisms, but rather as a sequential and mutually amplifying process in which chemical degradation enhances frost susceptibility, while frost damage further facilitates the ingress of aggressive ions.

Within this mechanistic framework, rubber particles are chemically inert and not directly affected by sulphate reactions. Under isolated chemical attack, their elastic deformability may locally accommodate limited volumetric expansion and reduce stress concentrations at low replacement levels [73,77,99]. Similarly, under freeze–thaw exposure alone, rubber inclusions can partially absorb ice crystallisation pressures during freezing and recover during thawing, potentially delaying internal damage accumulation and critical saturation levels in rubberised concretes [128,154,186].

However, the present results demonstrate that this potentially beneficial mechanism is only effective within a limited range of rubber contents. At high replacement levels, the increased porosity, previously evidenced by durability-related tests, facilitates deeper penetration of sulphate solutions and higher internal water saturation. Under these conditions, the elastic accommodation capacity of rubber particles becomes insufficient to counteract the combined effects of expansive sulphate reactions and repeated ice formation. Instead, the higher connectivity of the pore network promotes damage propagation, leading to extensive microcracking, loss of cohesion and severe strength degradation.

Overall, the results indicate that while low to moderate rubber contents (≤20%) may provide a degree of resistance under severe coupled exposure by partially accommodating internal stresses, higher replacement levels significantly exacerbate damage when freeze–thaw cycling and sulphate attack act simultaneously. This highlights the importance of considering combined environmental actions when assessing the durability of rubberised concretes and confirms that performance under isolated exposure conditions cannot be directly extrapolated to more aggressive service environments.

## 5. Conclusions

This study combined a structured literature review with an experimental investigation to clarify the performance and durability limits of rubberised concrete under coupled environmental actions.

The review of more than 150 published studies revealed a clear imbalance in existing research. Most investigations focus on mechanical properties, while durability-related performance remains comparatively underexplored, particularly for medium-to-high rubber replacement levels and under combined environmental exposures. In addition, the majority of previous studies adopt full fine-aggregate replacement strategies, with limited attention paid to the selective substitution of specific aggregate fractions within well-graded reference concretes. These gaps directly motivated the experimental programme developed in this study, particularly the adoption of a selective sand-fraction replacement strategy and the assessment under combined freeze–thaw and sulphate attack.

Based on the experimental results, the following conclusions can be drawn:

- The selective sand-fraction replacement strategy moderates the mechanical penalties commonly reported for rubberised concretes. Although mechanical properties decrease progressively with increasing rubber content, mixtures incorporating up to approximately 10–15% rubber exhibited compressive strength reductions limited to 15–20%, with a similar trend observed for flexural strength.
- Bulk density decreased progressively with increasing rubber content, reaching reductions close to 15% at the highest replacement level (50%). This reduction was accompanied by a significant increase in water-accessible porosity, exceeding 18% at high replacement levels (≥40%), which is typically associated with low durability performance. In contrast, for rubber contents below 15%, porosity increased only moderately, remaining around 13%, close to the value measured for the reference concrete (approximately 11%).
- The increase in porosity translated into reduced resistance to carbonation and chloride ingress. While concretes with low rubber contents (<15%) exhibited only limited increases in carbonation depth (approximately 10–20%) and chloride migration coefficients ranging from 2.86 × 10^−12^ m^2^/s up to 4.41 × 10^−12^ m^2^/s, which are associated with high durability performance, higher replacement levels led to pronounced reductions in resistance to both deterioration mechanisms.
- Electrical resistivity remained similar to or slightly higher than that of the reference concrete for rubber contents up to 20%, indicating that transport properties were not critically affected at these replacement levels. However, a sharp decrease in resistivity was observed when rubber content exceeded 30%, reflecting a substantial increase in pore connectivity and enhanced ionic transport capacity.
- Under combined freeze–thaw cycling and sulphate attack, mixtures containing 30% or more rubber exhibited severe surface damage and compressive strength losses exceeding 40–50%. In contrast, concretes incorporating low rubber contents exhibited negligible surface degradation and strength losses comparable to those of the reference concrete.

From an engineering perspective, the results indicate that rubber content must be carefully tailored to the intended performance requirements and exposure conditions. Within the selective sand-fraction replacement strategy adopted in this study, low to moderate replacement levels (≤15–20% of the targeted sand fraction) appear compatible with maintaining acceptable mechanical and durability performance, even under aggressive environmental conditions. In contrast, higher replacement levels (>30% of the substituted fraction) are associated with pronounced increases in porosity and transport properties, which may lead to significant reductions in strength and resistance to coupled deterioration mechanisms.

Overall, the study demonstrates that rubberised concrete can be technically viable when replacement strategy and service environment are considered jointly, thereby supporting a more rational and application-oriented approach to rubber valorisation in construction.

## Figures and Tables

**Table 1:** Mix proportions (kg/m^3^).

Cement	Water	SP	SG 4/12	SS 0/4	SS 0/2	LF	w/b
300	165	3.00	711	632	505	123	0.55

w/b: water/binder ratio.

**Table 2:** Rebound index and UPV tests’ results.

Rebound Index	CR-00	CR-05	CR-10	CR-15	CR-20	CR-30	CR-40	CR-50
-	28	26	24	25	26	20	17	19
(CV)	(1.85)	(2.66)	(1.55)	(1.34)	(1.10)	(1.77)	(1.55)	(1.95)
**UPV**	**CR-00**	**CR-05**	**CR-10**	**CR-15**	**CR-20**	**CR-30**	**CR-40**	**CR-50**
m/s	4350.48	4319.66	4087.89	4071.48	4040.44	3825.93	3720.94	3651.78
(CV)	(50.18)	(16.60)	(22.95)	(43.95)	(17.32)	(17.76)	(39.79)	(58.93)

CV: coefficient of variation.

**Table 3:** Carbonation depth.

Depth	CR-00	CR-05	CR-10	CR-15	CR-20	CR-30	CR-40	CR-50
mm	9.00	9.75	10.50	10.75	11.25	13.75	14.25	15.25
(CV)	0.76	1.07	0.76	0.76	0.46	0.64	0.64	0.71

CV: coefficient of variation.

## Data Availability

The raw data supporting the conclusions of this article will be made available by the authors on request.

## Abbreviations

The following abbreviations are used in this manuscript:

CDW	Construction and demolition waste
ELT	End-of-life tyre
RubC	Rubberised Concrete
r.p.	Rubber Particles
SF	Silica fume
SCM	Supplementary cementitious material
LF	Limestone Filler
w/b	Water/binder ratio
T	Temperature
RH	Relative Humidity
UPV	Ultrasonic pulse velocity
CV	Coefficient of variation
$D_{nssm}$	Non-steady state migration coefficient
